# Developing a psychosocial competency framework for adult and older adult acute mental health inpatient care

**DOI:** 10.1111/papt.12575

**Published:** 2025-02-14

**Authors:** Lisa Wood, Claire Williams, Leah Luxon, Ajvir Kumary, Anthony Roth

**Affiliations:** ^1^ Acute and Rehabilitation Directorate North East London NHS Foundation Trust, Goodmayes Hospital Essex UK; ^2^ University College London London UK

**Keywords:** competency framework, inpatient mental health care, psychosocial intervention

## Abstract

**Objectives:**

Acute mental health inpatient settings care for those with acute and complex mental health needs. This study aimed to develop a psychosocial competency framework for use by multidisciplinary professional practitioners working in adult and older adult acute mental health inpatient settings.

**Methods:**

The competence framework was developed through an iterative developmental process. Initially, the relevant literature was reviewed to draft the framework. A multidisciplinary Expert Reference Group provided their expertise to refine and develop the framework and provide professional advice on areas with a weaker evidence base.

**Results:**

A competency framework was produced and nine key areas of competency were developed; ‘Basic knowledge of issues related to acute mental health inpatient care’, ‘Communication skills’, ‘Professional competences for all healthcare workers’, ‘Generic relationship competences’, ‘Assessment, formulation and planning’, ‘Structured care and intervention’, ‘Psychosocial interventions’, ‘Meta‐competences’, and ‘Professional competences for organisations’.

**Conclusions:**

This competency framework will enable stakeholders to understand which competences are needed for high‐quality acute mental health inpatient care provision. It can be used to underpin training packages in this field. Our framework has been recommended as a best‐practice resource in the NHS England Acute Inpatient Mental Health Care guidance for adults and older adults.

## INTRODUCTION

Improving inpatient mental health care is a central issue for the National Health Service (NHS), as outlined by the recent Long‐Term Plan and Five Year Forward View for Mental Health (Independent Mental Health Taskforce, 2016; NHS England, 2019a). Acute mental health inpatient hospitals care for those with acute, severe, and enduring mental health difficulties who may be at high risk of harm to themselves or others (The Kings Fund, 2017). An increasing majority of admissions are compulsory detainments under the Mental Health Act (MHA). Mental health inpatient admissions are becoming briefer with the current average stay only being 31 days (NHS Benchmarking Network, 2019). Consequently, acute mental health inpatient settings are high pressured and high‐risk environments, making them potentially stressful environments to work in (NHS Benchmarking Network, 2019).

The severity and need of psychiatric inpatients and forced admissions is rising, indicating a systemic failure to provide effective crisis care for inpatients (Independent Mental Health Taskforce, 2016). A key recommendation by NHS England is to improve the delivery and quality of acute mental health crisis care by offering effective, safe, and compassionate care to patients. The government has also recently undertaken an independent review of compulsory MHA detention to understand why they occur and how to prevent them (HM Government, 2018). This indicated that inpatient care needs to restore the dignity of the patient, improve choice and decision making, and ensure that human rights are respected, and concluded that inpatient care delivery needs to be improved to achieve this.

Inpatient mental health care is underpinned by a biopsychosocial framework and delivered by a multidisciplinary team of mental health professionals (Bowers et al., 2009). Several initiatives have been developed to improve the delivery of inpatient care. For example, the Safewards model was developed to reduce conflict and increase containment by implementing 10 psychosocial interventions, such as calm‐down methods, and discharge messages of hope and recovery (Bowers et al., 2014). Moreover, the Royal College of Psychiatrists has published a set of standards for mental health inpatient settings which outlines the core components of care delivery for inpatient settings (Royal College of Psychiatrists, 2010). These standards outline criteria for admission and assessment, care planning and treatment, referral, discharge and transfer, patient and carer experience, staffing and training, environment and facilities, and governance. Whilst these strategies have been crucial in raising the quality‐of‐care delivery in inpatient settings, they do not outline the knowledge, skills, and attitudes (i.e. the competences), that staff require to work within inpatient settings. There is a need for an inpatient multidisciplinary psychosocial competency framework to ensure that inpatient care is delivered compassionately, holistically and safely, to a high standard by professionals working within this setting.

Competency frameworks aim to bring together the relevant knowledge, skills, and values that are required to work effectively in a specified clinical area (Roth & Pilling, 2012). They should be indicative rather than prescriptive, and require the use of clinical judgement (Roth & Donnan, 2019). They are a tool to support clinical practice and describe what would be expected of a competent clinician. A multidisciplinary competency framework can be used to inform training, supervision, and for individual practitioners to reflect on the quality of their practice. It has been widely acknowledged that the psychosocial component of inpatient care delivery needs significant improvement (Wood et al., 2019). Although psychosocial interventions are regularly cited by patients as crucial components of their inpatient care experience, they are often secondary to medical treatments (Wood et al., 2019). Therefore, the aim of this study was to develop a multidisciplinary psychosocial competency framework for use by professionals working in acute mental health inpatient settings following Roth & Pilling's best‐practice guidelines (Roth & Pilling, 2008). For the purposes of the framework, we defined psychosocial approaches as any methods or interventions that capitalise on psychological or social actions to produce a change in psychological, social, biological, and/or functional outcomes (England et al., 2015).

More specifically, the aims were:

1. To identify the knowledge, skills, and attitudes required to work in acute mental health inpatient settings.
2. To structure these competences in a way that makes them useful and accessible to the target audience.

## METHOD

### Design and ethics

This study followed competency framework development guidelines outlined by Roth and Pilling (2008), and reporting structure of another recently published competency framework (Roth & Donnan, 2019). The aim was to develop a user‐friendly competency framework which organised competences into a ‘map’. This structure has been applied across a wide range of competency framework domains including therapy‐specific frameworks, complex mental health problems, and risk behaviours (Parry et al., 2021; Pilling et al., 2018; Roth & Pilling, 2012). The map is designed to be viewed online in an interactive format so that each competency can be linked to a further list of more detailed competences. The competency framework was developed between August 2019 and March 2022. Ethical approval was not required as it did not involve any primary data collection.

### Competency framework development

#### Establishing and consulting the expert reference group (ERG)

An Expert Reference Group (ERG) is a panel of subject experts, which guide the development of the competency framework making key decisions on the structure and content of the framework. ERG members were national topic experts and were identified via the research group professional networks via publicly available academic or clinical profiles, and through UCL's patient involvement leads (to identify experience by experience). We ensured diversity in our expert‐by‐experience group by actively seeking out people from diverse backgrounds. The ERG comprised multidisciplinary researchers, clinicians, patients, and carers. Excluding the authors (who are a mixture of clinical and counselling psychologists and an assistant psychologist), we had four clinical psychologists, four experts by experience, three mental health nurses, three occupational therapists, and a consultant psychiatrist in the ERG.^1^ All were experts in the field of acute mental health inpatient care and had experience in inpatient care delivery and/or conducting research which informed practice in this setting. Other areas of relevant expertise were experiences of NHS management, academic leadership, and delivering training courses.

**FIGURE 1:**
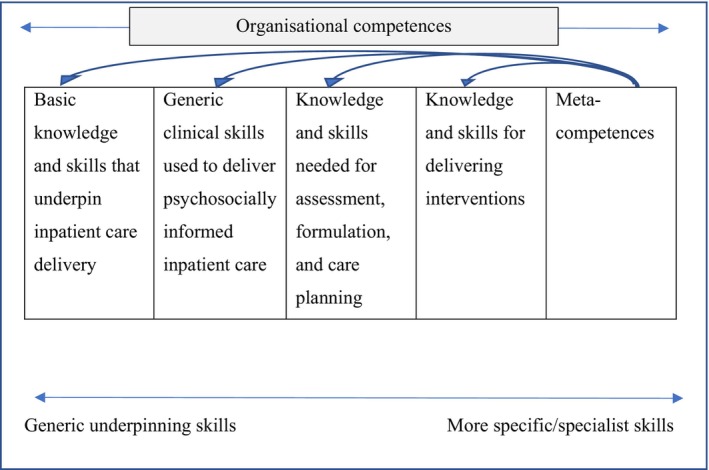
Outline structure of competence map.

The ERG met on three occasions (October 2019, January 2020, and November 2020) to refine the framework and to give feedback. The first meeting was spent reviewing literature identified by the research team, identifying any gaps in the literature, and beginning to review core parts of the framework (specifically the basic knowledge and skills and generic clinical skills – see Figure 1). The second meeting was spent reviewing the knowledge and skills needed for assessment formulation and care planning, knowledge, and skills for delivering interventions, and organisational competences sections. The final meeting was spent reviewing meta‐competences and the overall final draft of the framework. Every meeting was chaired by a member of the research group who ensured that all stakeholders were able to share their perspectives on the framework development. When there were diverging opinions, consensus was achieved in the meetings through detailed discussion and debate.

#### Gathering relevant literature

Firstly, several sources of information were drawn upon to develop the competency framework. These were identified by the research group which collated resources relating to the interdisciplinary psychosocial delivery of acute mental health inpatient care; the ERG also suggested further key resources in the first ERG meeting. This led to a variety of materials being included: key policies and clinical guidelines (Care Quality Commission, 2016; Ebrahim & Wilkinson, 2021; HM Government, 1983, 2005; National Collaborating Centre for Mental Health, 2009; National Institute of Health and Care Excellence, 2003; NHS England, 2019a, 2019b; NICE, 2014; Royal College of Psychiatrists, 2015, 2016, 2019), seminal research papers on the delivery of inpatient care (Bindman et al., 2003; Bowers et al., 2009; Bowers et al., 2014; Muskett, 2013; Royal College of Psychiatrists, 2010; The Commission on Acute Adult Psychiatric Care, 2015; Wykes et al., 2018), existing relevant competency frameworks (Pilling et al., 2018; Pilling & Roth, 2021; Roth & Pilling, 2008, 2012), systematic reviews relevant to acute inpatient care delivery and research that included the lived experience perspective (Hamrin et al., 2009; Reen et al., 2020; Wood & Alsawy, 2016).

#### Developing an initial draft of the framework

Initial competences were developed from these sources. Items or information were extracted from these above documents if they were deemed to reflect knowledge, skills, or attitudes key to the delivery of inpatient settings, adapting these to fit the format of the competency framework. There are limitations to this stage because source documents which explicitly outline competences can be limited. Whilst there are manuals that detail explicit competences in delivering interventions, more often the literature is presented at a more conceptual level and requires the researcher to adapt the information into competences. For example, the Royal College of Psychiatrists risk assessment document lists key risk behaviours (for example, self‐harm, suicide, violence and aggression, abuse, neglect, exploitation) that clinicians should be knowledgeable of (Royal College of Psychiatrists, 2016); this was turned into a competency framework item by rewording it to “An ability to draw on knowledge of definitions of key risk behaviours (for example, self‐harm, suicide, violence and aggression, abuse, neglect, exploitation)”. Competency items were grouped together if they were deemed to reflect a similar competency, and then organised into a ‘map’ which gives an overview of the key areas of knowledge, skills, and attitudes. Figure 1 outlines the basic structure of the framework and is adapted from Roth and Donnan (2019). Roth and Donnan (2019) used the same structure and had five categories of: basic knowledge and skills, generic clinical skills, knowledge, and skills needed for assessment formulation and care planning, knowledge, and skills for delivering interventions and meta‐competences. We tailored these to the inpatient setting and added a sixth category of “organisational competences”. The final six areas of our framework included core knowledge and skills, generic clinical skills, knowledge and skills required for assessment, formulation and care planning, knowledge and skills for delivering interventions, meta‐competences (the procedural knowledge that practitioners need if they are to implement competences flexibly), and competences for organisations. These are explored in more detail in the results section.

## RESULTS

**FIGURE 2:**
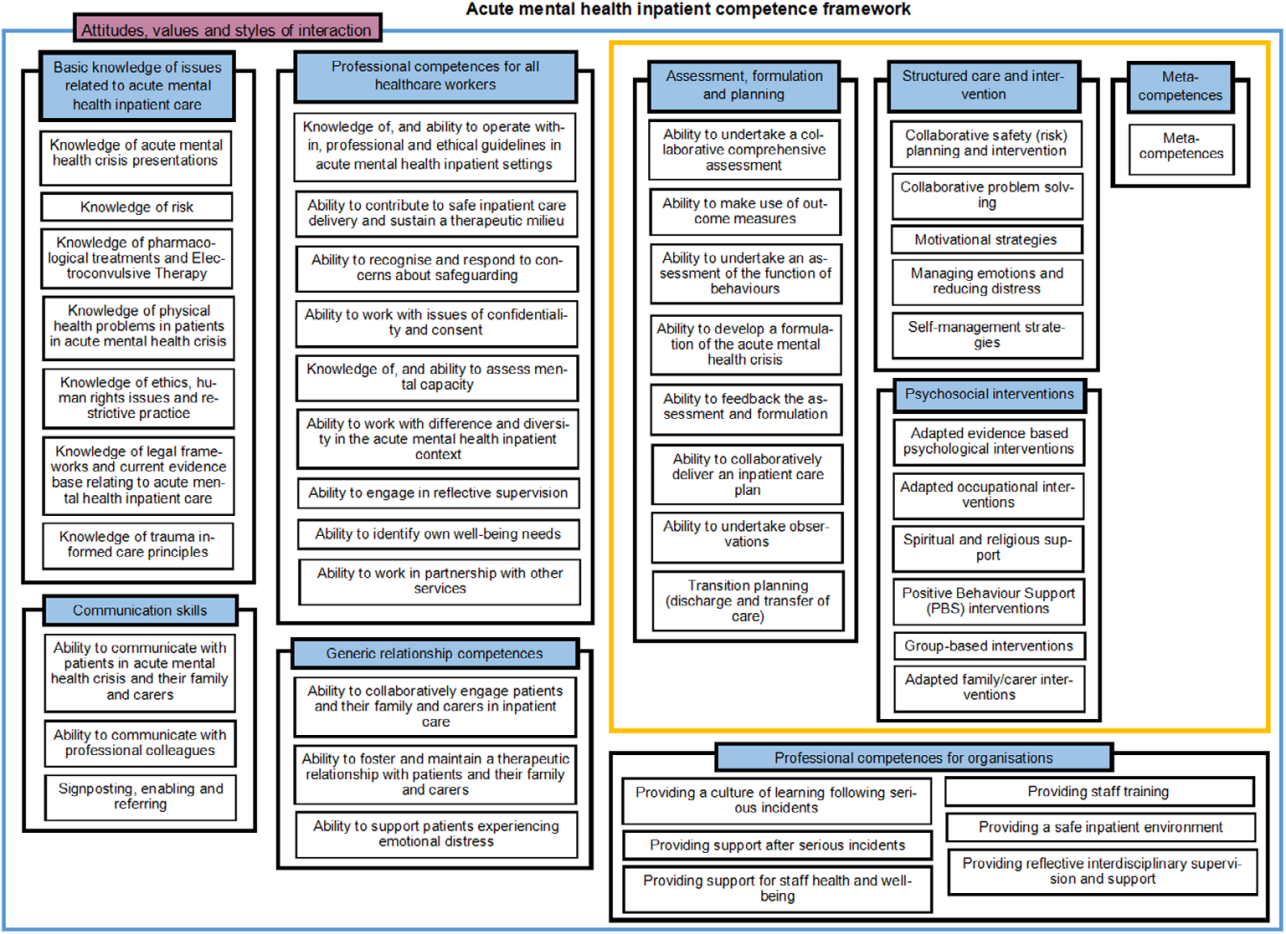
Acute mental health inpatient competency framework.

The map of acute menta health inpatient competences can be found in Figure 2. The interactive map and full list of the competences can be found on the UCL competency framework website (https://www.ucl.ac.uk/pals/research/clinical‐educational‐and‐health‐psychology/research‐groups/competence‐frameworks). Below is a summary of the framework's content.

### Attitudes, values, and style of interaction competences

**FIGURE 3:**
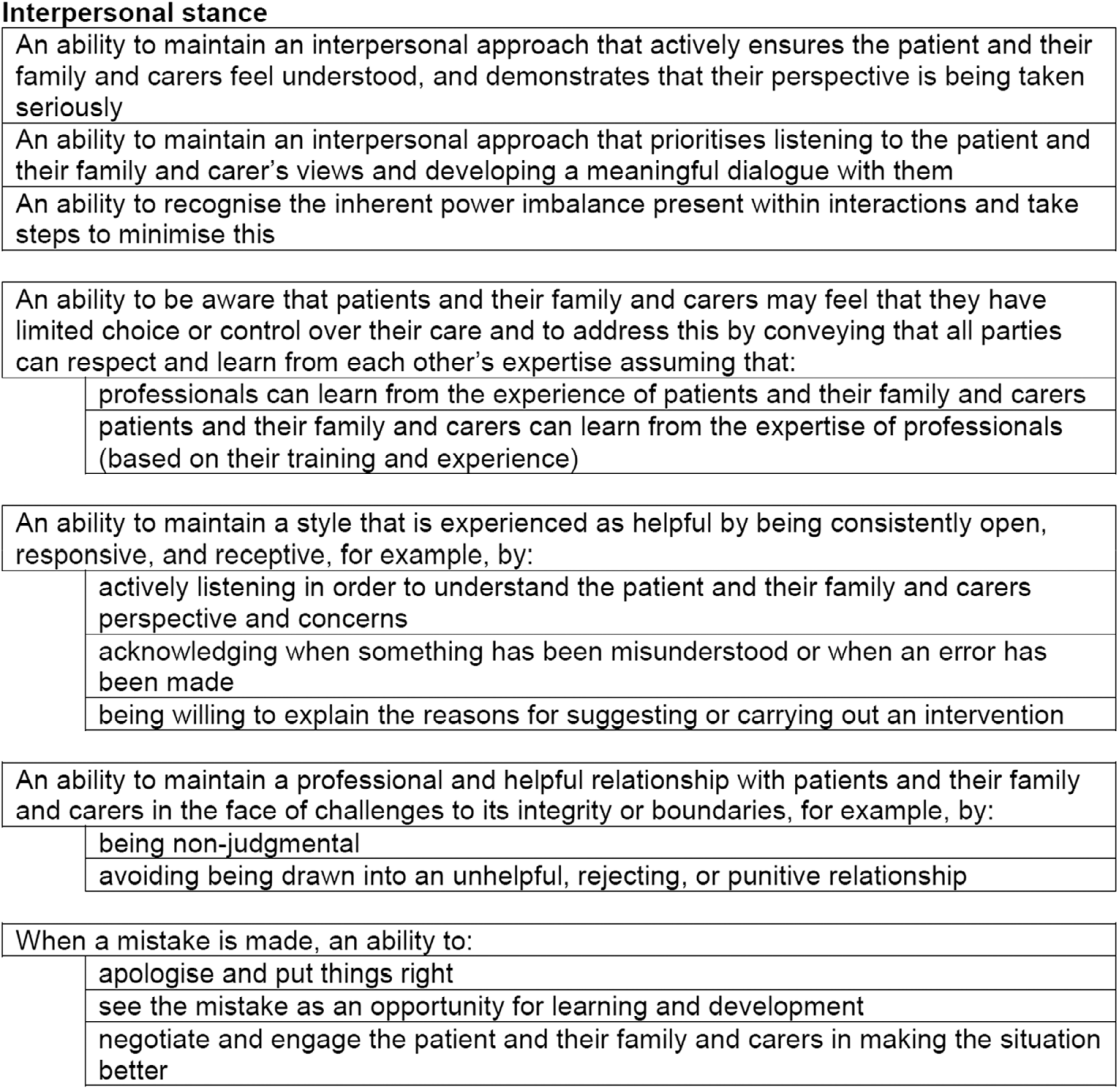
Attitude, values, and styles of interaction example competences.

The first component of the framework describes the attitudes, values, and style of interaction when working with patients in acute mental health inpatient settings. These competences underpin the whole competency framework and therefore are important to all aspects of care delivery. Examples of competence items in this section can be found in Figure 3. This section emphasises that work with patients and their family and carers must be underpinned by a compassionate, respectful, and non‐judgemental human relationship. Inpatient care takes place in a restrictive and disempowering environment with marginalised populations (i.e. patients from Black African and Caribbean backgrounds) over‐represented in this setting, and practitioners need to be mindful of the inherent power imbalances that occur between professionals and patients, particularly when the patient is compulsorily detained. This set of competences outline ways of addressing this power imbalance, treating patients and their support network respectfully and compassionately, empowering patients, and working from a person‐centred position. This requires professionals to understand a patient's difficulties within their social and cultural context, ensuring that they feel that their freedoms and rights are always respected, and have as much control as possible over their care, even when detained.

### Basic knowledge of issues related to acute mental health inpatient care

This part of the framework focuses on the knowledge required to deliver acute mental health inpatient care. The section ‘Knowledge of acute mental health crisis presentations’ outlines the key characteristics of an acute mental health crisis and the common reasons for admission. ‘Knowledge of risk’ outlines the common risk behaviours which occur in crisis presentations. The competences contained within ‘Knowledge of pharmacological treatments and Electroconvulsive Therapy (ECT)’ highlight the function of these interventions and the knowledge needed to prescribe medication and ECT. ‘Knowledge of physical health problems in patients in acute mental health crisis’ highlights the importance of physical healthcare provision in inpatient settings and the problems of diagnostic overshadowing and its links with health inequalities.

‘Knowledge of ethics, human rights and restrictive practice’ is integral to care delivery in this setting as human rights are at risk of being compromised. This includes knowledge regarding de‐escalation strategies and how to reduce the use of restrictive practices. ‘Knowledge of legal frameworks and current evidence base relating to acute mental health inpatient care’ reflects the importance of professionals being knowledgeable about relevant legislation and frameworks to provide safe care, such as whistleblowing procedures and the Mental Health Act (MHA). This is key to working in this area, as knowledge of mental health law and issues such as consent and capacity are part of routine practice. Finally, ‘Knowledge of trauma‐informed care’ describes core knowledge about how inpatient services can be trauma‐informed, provide sensitive and safe care, and not retraumatise patients.

### Communication skills

**FIGURE 4:**
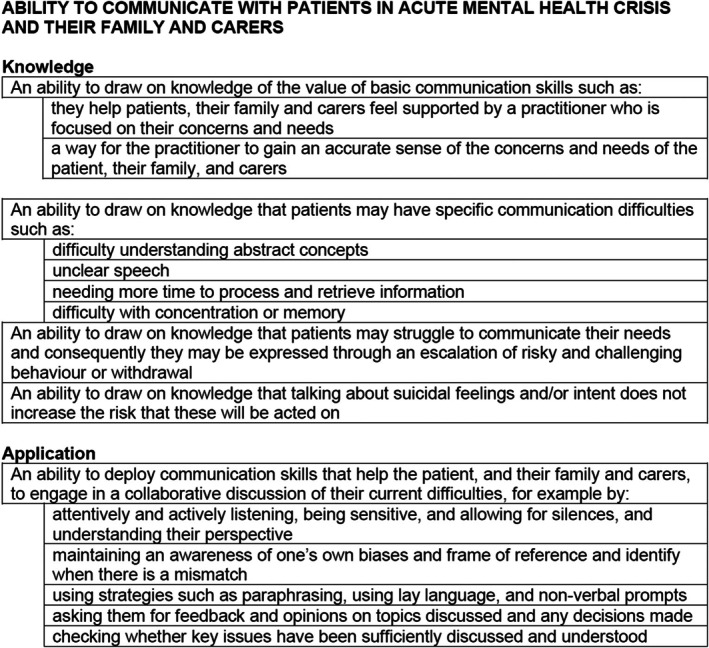
Communication skills example competences.

Communication skills competences are outlined in Figure 4. ‘An ability to communicate with patients in acute mental health crisis and their family and carers’ is fundamental to care delivery to ensure they feel heard and understood, can express themselves in their own words, and are involved in all care decisions. ‘An ability to communicate with professional colleagues’ allows efficient delivery of high‐quality multidisciplinary holistic care provision. Finally, in this part of the framework, the role of ‘Signposting, enabling and referring’ is outlined, setting out the competences needed to direct patients to resources and sources of support and support uptake.

### Professional competences for all healthcare workers

All professions and regulatory bodies outline ethical standards that professionals are expected to operate within, and the competences within ‘Knowledge of, and ability to operate within, professional and ethical guidelines’ reflect this. ‘Ability to contribute to safe inpatient care delivery and sustain a therapeutic milieu’ maps out how individual practitioners can contribute to a safe and therapeutic inpatient culture, which minimises the use of restrictive practices. To protect individuals who are at risk of harm from various forms of abuse or neglect, professionals should have an ‘Ability to recognise and respond to concerns about safeguarding’. ‘Knowledge of, and ability to work with, issues of confidentiality and consent’ outlines judgements about when (and to whom) it is in the patient's best interests to maintain or to breach confidentiality. Related to this is ‘Knowledge of, and ability to assess, capacity’ which should be considered for all patient decision‐making.

Respecting diversity, promoting equity, and challenging discrimination, are critical aspects of practice and are outlined in the ‘Ability to work with difference and diversity in the acute mental health inpatient context’ section. All professionals should be able to care for patients from all backgrounds, including those with protected characteristics, or additional characteristics that might be relevant, such as socioeconomic status. Supervision and support for professionals should be the norm, so the ‘Ability to engage in reflective supervision’ is an essential competency to incorporate in this framework, particularly due to the highly stressful and emotive nature of inpatient settings. Relatedly, it is important for professionals to have the ‘Ability to identify own well‐being needs’ to ensure they can seek appropriate support when needed. Finally, the ‘Ability to work in partnership with other services’ is important for continuity of care across services.

### Generic relationship competences

Developing a therapeutic alliance and empowering patients is a key priority in inpatient settings. Professionals should have the “Ability to collaboratively engage patients and their family and carers in holistic evidenced based treatments”. Developing the alliance depends on an ability to recognise the ways in which patients and their families and carers understand themselves and the world around them, as well as their own goals, strengths, and needs. This makes the ‘Ability to foster and maintain a good therapeutic relationship with patients and their family and carers’ an essential area of skill. The ‘Ability to help patients experiencing emotional distress’ is central to all interactions with a person. Professionals should be able to help patients manage their emotional distress, which is likely to be heightened in this setting.

### Assessment, formulation, and planning

**FIGURE 5:**
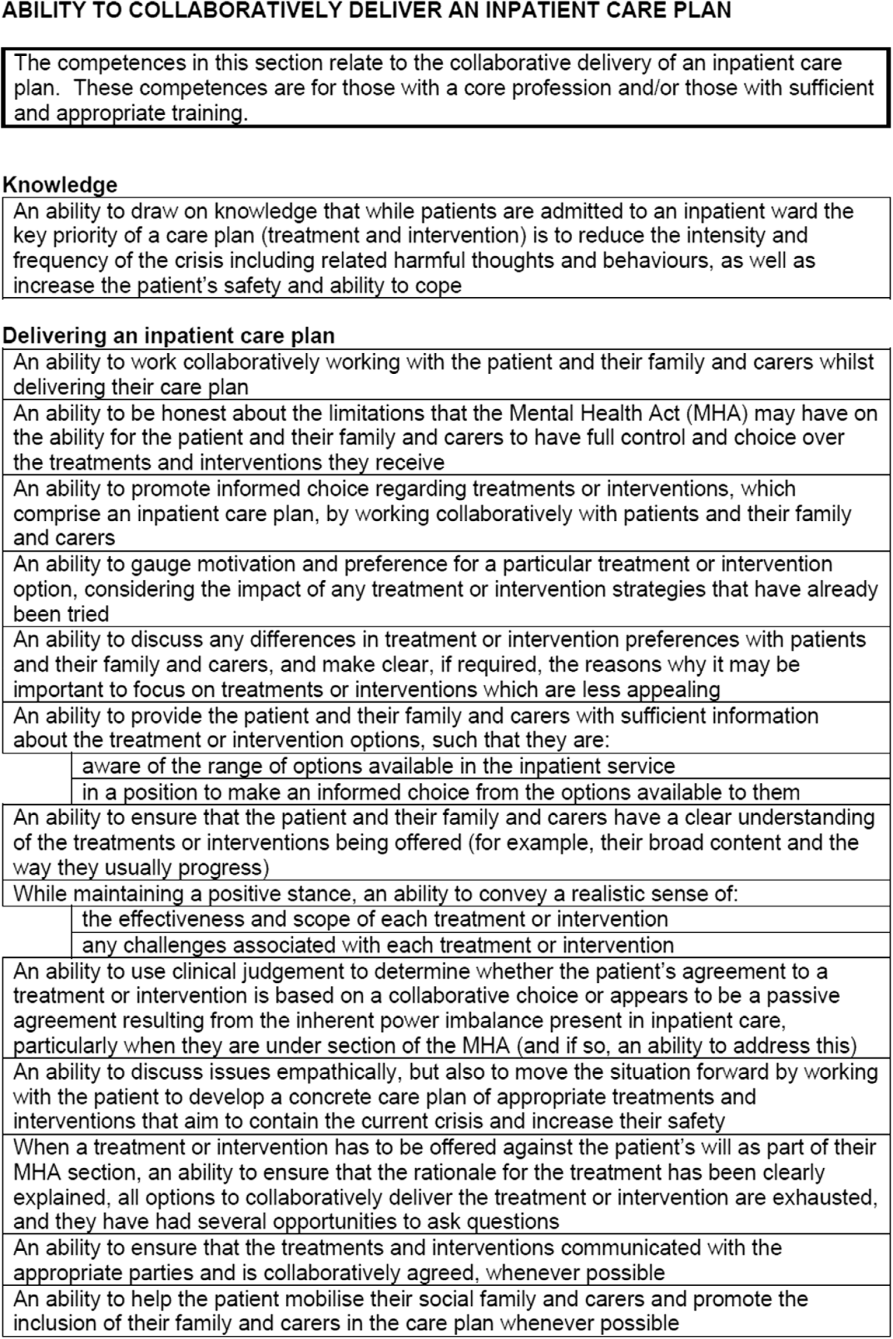
Assessment, formulation and planning example competences.

Exemplar competences are in Figure 5. It is important for professionals to have the ‘Ability to undertake a comprehensive assessment’ that considers a patient's safety within the context of their needs, also recognising the limitations of an assessment, particularly regarding risk. It is good practice for professionals to have the ‘Ability to make use of outcome measures’ so that changes in patients' mental health can be recorded systematically. Professionals should have the ‘ability to undertake an assessment of the function of behaviours'. Patients may present with behaviours that challenge and that may require further observational and specialist assessment. The ‘Ability to develop a formulation of the acute mental health crisis' is a key step in the assessment process, as this is the point at which information is gathered into a coherent account that helps to understand the determinants of a patient's crisis experiences. Following this, professionals should have the ‘Ability to feedback the assessment and formulation’ in a collaborative and sensitive manner. An ‘Ability to collaboratively deliver an inpatient care plan’ is the final but essential part of the process. Professionals should engage the patient (and their family and carers) in collaboratively exploring treatment options, even when receiving care under the MHA. ‘An ability to undertake observations’ is an important assessment activity that can maintain the safety of patients at risk of harm and should be seen as part of the clinical intervention rather than a stand‐alone, ‘tick‐box’ exercise. Transitions in care, including discharge from the ward, represent periods of risk for patients, particularly those who present with self‐harm or suicidal behaviours. For this reason, ‘Transition planning (discharge and transfer of care)’ should be anticipated and carefully considered. Professionals should coordinate the transition with the receiving service, support the patient in transitioning, and monitor the success of the transition.

### Structured care and intervention

Patients in inpatient settings usually have an array of complex needs that need to be responded to and managed in a time‐limited fashion. This often involves drawing upon a range of brief interventions to help increase safety and address their needs. ‘Collaborative safety (risk) planning and intervention’ is a crucial part of inpatient care as threats to safety/risk behaviours are often the primary reason for admission. When patients are in crisis, they often face a multitude of difficulties that they are struggling to manage. Therefore, ‘Collaborative problem solving’ can be an important intervention to help patients manage key problems contributing to and maintaining their crisis. Inpatients may also benefit from ‘motivational strategies’ when they are overwhelmed with hopelessness and suicidal ideation or struggling to overcome drug and alcohol misuse, which may be perpetuating their crisis. Mental health crises are often characterised by high levels of emotional distress and supporting patients with ‘emotional regulation and reducing distress’ may be an important intervention to increase a patient's sense of safety. However, strategies should be utilised in a manner that does not invalidate a patient's distress. Finally, self‐management strategies are important interventions for crisis management. Professionals should support patients to develop their autonomy and manage their own crisis, which should include strategies such as psychoeducation, coping strategy engagement, and relapse prevention.

### Psychosocial interventions

**FIGURE 6:**
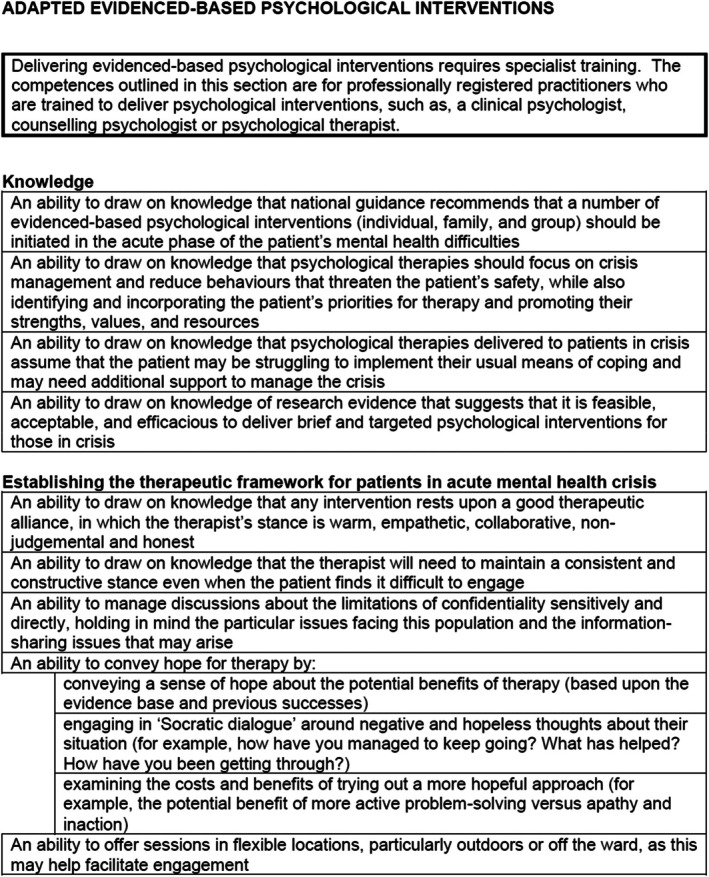
Psychosocial interventions example competences.

Exemplar competences can be found in Figure 6. Multidisciplinary psychosocial interventions are essential components of inpatient care. Patients want access to a wide range of psychosocial activities when admitted to a ward (Wood & Alsawy, 2016). As inpatient admissions are brief and within the context of a restricted environment, psychosocial interventions need to be focused on managing the presenting crisis and increasing safety. Patients should receive ‘adapted evidenced‐based psychological interventions’ which support them with their crisis experiences and increase their feelings of safety. There is a limited evidence base for psychological therapies in this setting, but there is some evidence that Cognitive Behaviour Therapy (second and third‐wave approaches) may be useful (Jacobsen et al., 2018). ‘Adapted occupational interventions’ are also important as occupational difficulties can be a key characteristic of an acute mental health crisis. Patients may require ‘spiritual and religious’ support during their time on the ward. Spirituality and religion are important frameworks for understanding and coping with mental health crisis for many patients. ‘Positive Behavioural Support (PBS) interventions’ are important when a patient presents with behaviours that challenge those around them. An integral component of inpatient care delivery is the provision of a comprehensive interdisciplinary group programme. ‘Group‐based interventions’ should be underpinned by a therapeutic model and offer opportunities for coping and interpersonal skill development, and peer support. Family and carers often find having their relative in a hospital an understandably difficult experience and may need support themselves or assistance to support their relative. Therefore, ‘adapted family/carer interventions’ should be offered to patients and their support network during an inpatient admission.

### Meta‐competences

**FIGURE 7:**
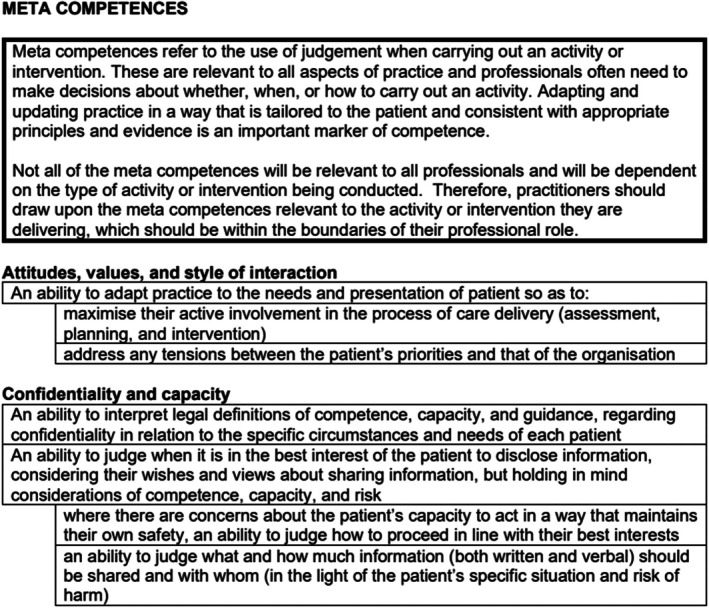
Meta‐competences.

The next section of the framework identifies overarching meta‐competences, which refer to the use of judgement when carrying out an activity or intervention (see examples in Figure 7). These are relevant to all aspects of practice, and professionals often need to make decisions about whether, when or how to carry out an activity. Adapting and updating practice in a way that is tailored to the person and consistent with appropriate principles and evidence is an important marker of competence.

### Professional competences for organisations

**FIGURE 8:**
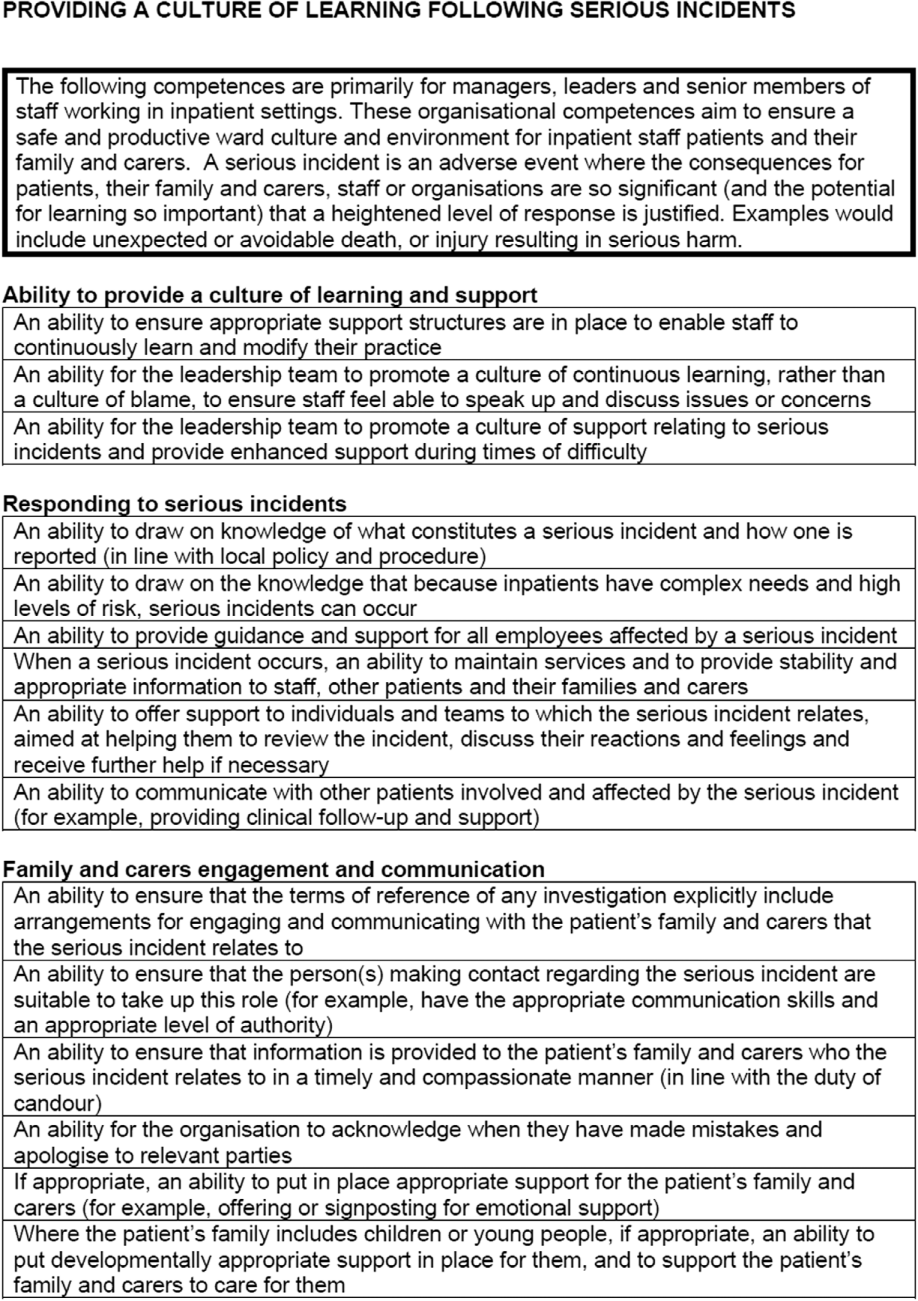
Professional competences for organisation example competences.

Because inpatient settings are highly complex and challenging work environments, organisational competences – and hence organisational support – are essential to the delivery of a safe environment for staff, patients and their family and carers. Example competences can be found in Figure 8. The first set of competences in this group reflects the importance of ‘Providing a culture of learning following serious incidents’, which involves setting up an independent investigation into serious incidents which adopts a non‐blaming culture and attempts to ensure that incidents are learnt from, and practice is continuously improved. Relatedly, ‘Providing support after serious incidents’, outlines the support required for staff who are impacted by a serious incident. Another key organisational competency is ‘Providing support for staff health and well‐being’, reflecting the fact that inpatient care settings have some of the highest rates of staff burnout, sickness and stress, and organisational support is required to address this. The next set of competences relates to ‘Providing staff training’. For staff to be competent in delivering care to patients with complex health needs, staff need regular and up‐to‐date training provision. This improves the quality of care provided and ensures that staff feel valued and confident in care delivery. ‘Providing a safe inpatient environment’ outlines key factors which ensure the inpatient context can operate safely, which relate to physical safety, safe staffing numbers, and ongoing service improvement. Finally, ‘Providing reflective interdisciplinary supervision’ outlines the importance of multidisciplinary group spaces where professionals can learn and reflect with one another. This is important for service quality, improving staff‐patient relationships, and facilitating a culture of learning.

## DISCUSSION

This article outlines the development of a competence framework for the delivery of psychosocial interventions in adult and older acute mental health inpatient settings. To the best of our knowledge, this is the first framework to be developed and applied specifically to this setting; it describes the key areas of attitudes, knowledge, and skills required of practitioners, with the aim of improving the quality of care. Our framework has been recommended as a resource for best practice in the recent NHS England Acute Inpatient Mental Health Care guidance for adults and older adults (NHS England, 2023).

The framework prioritises the development of the therapeutic relationship that should be characterised by empowerment, respect, safety, and inclusion, a factor highlighted by ERG members with lived experience. This reflects research studies which have demonstrated that the relationship with staff and peers is fundamental to inpatient care delivery (Moreno‐Poyato et al., 2016). Moreover, the ERG was clear that the framework should address the fact that inpatients are often from marginalised populations. As such we attempted to ensure that the framework encompasses the competences required for staff to work with experiences of trauma, marginalisation, racism, and discrimination. Strong organisational leadership is key to the effective delivery of inpatient care (Care Quality Commission, 2022), and the ERG was clear that competences for organisations should be included in the framework. Closed cultures can easily develop in inpatient settings, and strong leadership which prioritises staff and patient safety and supports the provision of high‐quality care is key to preventing such cultures from developing (Care Quality Commission, 2020). Another important inclusion in the framework are competences required for delivering interventions specifically tailored to the inpatient setting. Whilst there is reasonable evidence for assessment and formulation and risk management in inpatient settings, the evidence for inpatient‐adapted interventions is underdeveloped (Raphael et al., 2021). We have drawn on the best available evidence and utilised expertise from the ERG to outline competences for structured care and intervention and psychosocial intervention delivery.

The framework has several strengths. Firstly, as far as we are aware it is the first competency framework which has been developed to underpin the delivery of acute inpatient care from a psychosocial perspective. Moreover, it was developed with multidisciplinary expertise, including people with lived experience of inpatient care. It is also available as an open‐access document, free to use across the UK and internationally. The framework also went through a robust iterative process with several stages on input from the ERG demonstrating rigour.

There are limitations to the framework. First, it has been developed in the UK and may not reflect care delivery in other countries. Although there are similarities across settings internationally, the lack of international collaborators and the primary inclusion of ERG members who work in UK settings will mean that it will not be fully applicable to other international contexts. Further refinements will be required to make this framework suitable to other countries. Another limitation is the lack of rigorous and systematic processes for the development of the framework. Although we followed existing competency framework development processes (e.g. Roth & Donnan, 2019), these did not involve systematic processes (e.g. systematic reviews) or formal consensus methods (e.g. Delphi studies) meaning bias may be present. A recent competency framework scoping review identified that there is no consensus on how competency frameworks are developed and that significant variation is present (Batt et al., 2020). Furthermore, we did not include methods such as practice analysis which is an approach that examines the tasks performed by practitioners and the knowledge required for competent performance. This approach is often used in competency framework development to inform content, which may mean key competences have been omitted (National Board of Certification in Occupational Therapy, 2022). Moreover, the framework did not cover all aspects of acute mental health inpatient care delivery such as system mapping and capacity building. It was beyond the scope of the framework to include all aspects of inpatient care delivery relevant to all professionals' groups but may mean key areas are omitted. This framework, and all competency frameworks, should be reviewed at regular intervals and updated to ensure they reflect current best practices. To the authors knowledge, there are no best‐practice guidelines regarding reviewing and updating competency frameworks. However, we recommend that all competency frameworks should be reviewed approximately every 5–10 years based on research lifecycles and policy development timeframes. In the absence of an existing process, we recommend that a competency framework review should include a systematic search of newly published relevant literature in the field and establishment and consultation with an ERG to consider how the framework should be updated. Finally, we strived to have a diverse ERG group and managed to recruit lived experience members from diverse backgrounds however we could have included more experts by experience members and had more diversity in our group members.

This framework has several important applications. Firstly, the framework lends itself to the development of curricula for multidisciplinary inpatient professionals, due to its logical structure covering basic, specific, and specialist competences. Inpatient settings are challenging workplaces with high levels of staff turnover (Adams et al., 2021); therefore the development of a comprehensive training package utilising this framework would significantly improve inpatient care quality, staff retention, and patient experience. The framework could be used in supervision by both the person receiving the supervision and the supervisor. It can be used as a tool to guide and reflect upon their professional practice. A combined training and supervision package underpinned by the framework would ensure that improvements to practice are maintained in longer‐term care delivery, as research has shown that one‐off training packages do not sustain improvements or changes in the quality of inpatient care. Sustained changes to care cannot be achieved by training alone and system changes, such as long‐term supervision, are required to ensure long‐term behavioural change (Health Education England, 2016).

The framework could also be used by commissioners to ensure that the services they commission meet the competences. The framework could guide the distribution of resources by ensuring services have the appropriate skill mix to meet the local population's needs. Services can use this framework to evaluate their current practice and processes. This may help inform the safer delivery of care and increase its quality. Finally, the framework could also be used to inform research studies and audits and benchmark the degree to which services comply with the competences.

In conclusion, this psychosocial competency framework identifies the attitudes, knowledge, and skills required of professionals to deliver safe and effective inpatient care. It aims to tackle important issues within inpatient care such as ensuring it is trauma‐informed, supports patients with social difficulties, and is applicable to ethnic minority patients over‐represented in this setting. As such it has important clinical implications for this setting and can be used to inform training, care delivery, evaluations, personal practice and commissioning.

## AUTHOR CONTRIBUTIONS

**Lisa Wood:** Conceptualization; writing – original draft; methodology; writing – review and editing; project administration; supervision. **Claire Williams:** Conceptualization; writing – review and editing; methodology. **Leah Luxon:** Writing – review and editing; methodology; investigation. **Ajvir Kumary:** Methodology; writing – review and editing; investigation. **Anthony Roth:** Methodology; writing – review and editing; supervision; conceptualization; investigation.

## CONFLICT OF INTEREST STATEMENT

There are no conflicts of interest.

## Data Availability

Data availability is not applicable to this article as no new data were created or analysed in this study.

## Footnotes

^1^
Members of the ERG were Katherine Berry, Mary Birken, Selma Ebrahim, Jarka Hinksam, Pamela Jacobsen, Ajvir Kumary, Winnie Liu, Leah Luxon, Kieran Mahoney, Fiona Nolan, Patrick Nyikavaranda, Stuart Reid, Anthony Roth, Abdi Sanati, Alan Simpson, Susan Smythe, Paul Ware, Claire Williams, Suzie Willis, and Lisa Wood. Additional consultation was sought from Mina Patel to ensure it was compatible with older adult inpatient settings.
